# Interaction of systemic oxidative stress and mesial temporal network degeneration in Parkinson’s disease with and without cognitive impairment

**DOI:** 10.1186/s12974-018-1317-z

**Published:** 2018-09-26

**Authors:** Pi-Ling Chiang, Hsiu-Ling Chen, Cheng-Hsien Lu, Yueh-Sheng Chen, Kun-Hsien Chou, Tun-Wei Hsu, Meng-Hsiang Chen, Nai-Wen Tsai, Shau-Hsuan Li, Wei-Che Lin

**Affiliations:** 1 Department of Diagnostic Radiology, Kaohsiung Chang Gung Memorial Hospital, Chang Gung University College of Medicine, 123 Ta-Pei Road, Niao-Sung, Kaohsiung, 83305 Taiwan; 2 Department of Neurology, Kaohsiung Chang Gung Memorial Hospital, Chang Gung University College of Medicine, Kaohsiung, Taiwan; 3 Brain Research Center, National Yang-Ming University, Taipei, Taiwan; 4 Department of Radiology, Taipei Veterans General Hospital, Taipei, Taiwan; 5 Department of Hematology and Oncology, Kaohsiung Chang Gung Memorial Hospital, Chang Gung University College of Medicine, Kaohsiung, Taiwan

**Keywords:** Parkinson’s disease, Systemic oxidative stress, Lymphocyte apoptosis, Mesial temporal network, Gray matter volume, Functional connectivity, Cognitive impairment

## Abstract

**Background:**

To identify the vulnerable areas associated with systemic oxidative stress and further disruption of these vulnerable areas by measuring the associated morphology and functional network alterations in Parkinson’s disease (PD) patients with and without cognitive impairment.

**Methods:**

This prospective study was approved by the institutional review board of KCGMH, and written informed consent was obtained. Between December 2010 and May 2015, 41 PD patients with different levels of cognitive functions and 29 healthy volunteers underwent peripheral blood sampling to quantify systemic oxidative stress, as well as T1W volumetric and resting state functional MRI (rs-fMRI) scans. Rs-fMRI was used to derive the healthy intrinsic connectivity patterns seeded by the vulnerable areas associated with any of the significant oxidative stress markers. The two groups were compared in terms of the functional connectivity correlation coefficient (fc-CC) and gray matter volume (GMV) of the network seeded by the vulnerable areas.

**Results:**

The levels of oxidative stress markers, including leukocyte apoptosis and adhesion molecules, were significantly higher in the PD group. Using whole-brain VBM-based correlation analysis, the bilateral mesial temporal lobes (MTLs) were identified as the most vulnerable areas associated with lymphocyte apoptosis (*P* < 0.005). We found that the MTL network of healthy subjects resembled the PD-associated atrophy pattern. Furthermore, reduced fc-CC and GMV were further associated with the aggravated cognitive impairment.

**Conclusion:**

The MTLs are the vulnerable areas associated with peripheral lymphocyte infiltration, and disruptions of the MTL functional network in both architecture and functional connectivity might result in cognitive impairments in Parkinson’s disease.

**Electronic supplementary material:**

The online version of this article (10.1186/s12974-018-1317-z) contains supplementary material, which is available to authorized users.

## Background

Parkinson’s disease (PD) is the second most common neurodegenerative movement disorder among the elderly and also presents as a spectrum of cognitive dysfunction, ranging from PD with normal cognition (PDN) to PD with mild cognitive impairment (PDMCI) to PD with dementia (PDD) [1]. PDMCI is an early form of neurodegeneration that carries a risk of further degeneration into dementia. It is now known that early cognitive deficits constitute an important issue for diagnostic, therapeutic, and prognostic factors in PD [1]. In previous studies, dementia in PD has been demonstrated to be associated with widespread gray matter (GM) atrophy [2], especially in the mesial temporal lobe (MTL) [3]. Although the brain is less affected in cases of PDMCI than dementia, the initial atrophy patterns seen in PDMCI can be used for dementia prediction [3, 4].

In addition to structural changes, alterations of both dopaminergic and non-dopaminergic transmitter systems in the PD brain have also been found to modify several distinct functional networks underlying the different cognitive impairments [3]. The functional network disruptions assessed with “resting state” or intrinsic connectivity fMRI has shown distinct patterns of global network disruption or disruption within specific networks in PD in recent studies. Different network connectivity changes affect different types of cognitive impairment in PD Different network connectivity changes result in different types of cognitive impairment in PD [5–8]. Progressive cognitive decline in PD is associated with altered resting status functional connectivity in multiple brain regions, including the MTL [9]. Several studies have also discussed dopamine-dependent functional connectivity disruptions and other network alternations in PD [10]. Furthermore, a previous review study has shown worsening memory storage deficits associated with worsening MTL atrophy and further damage to the MTL network with the progression from PD to PDD. Another network implicated in memory is the basal nucleus of Meynert (BNM) cholinergic network. The volume of the BNM cholinergic network degenerates significantly in PDD, which correlates with progressive electrocortical depression in magnetoencephalography [3].

In the large-scale study of human brain networks by Seeley et al., it was reported that spatial disease atrophy patterns reflect the healthy brain’s intrinsic functional network architecture. These findings support the network neurodegeneration hypothesis of Seeley et al. [11] and suggest that human neural networks can be defined by synchronous baseline activity and selective vulnerability to neurodegenerative illness. However, while these functional networks are associated with cognitive dysfunctions, the specific factors that affect the vulnerable areas, and their locations and contributions to the degeneration from mild cognitive impairment to dementia in PD are still undetermined.

Systemic oxidative stress is an important etiology of the neuroinflammation seen in PD and is associated with further progression of the disease via various pathways, such as blood-brain barrier (BBB) dysfunction and the infiltration of peripheral immune cells and circulating cytokines [12, 13]. The infiltration of peripheral immune cells, especially lymphocytes and monocytes, which can mediate apoptosis [14], is one of the interactive pathways of systemic oxidative stress and neuroinflammation. The migration of lymphocytes to the central nervous system (CNS) through the BBB is dependent on lymphocyte function-associated antigen 1 (LFA-1) [15]. This association has been further been demonstrated by the increased peripheral leukocyte apoptosis with striatal dopamine neuron loss [16] and white matter alteration observed through the use of diffusion tensor imaging [17]. In addition, increased oxidative stress has been found to be associated with cognitive function status even in the early stage of the disease in young PD patients [18]. The accumulating evidence has shown an association between inflammation and cognitive impairment not only in PD [19] but also in other neurodegenerative diseases such as AD [20] and even in changes due to normal aging [21]. Peripheral immune cells, which reflect the level of systemic oxidative stress, might play important roles both in disease progression and cognitive function deterioration in PD.

The main focus with respect to peripheral immune cells was on their role in dopamine neuron loss in the substantia nigra (SN) or striatum in previous studies. Although the peripheral immune cells are associated with the initial pathogenesis of PD [22], it is still not known whether this association is widely spread in a non-specific manner to all brain tissues or if it is only focused on particular regions, i.e., the so-called vulnerable areas. Some recent studies have demonstrated the correlational relationships between structural alterations of various areas and different peripheral oxidative stress markers [17, 18, 23]. Moreover, two mediation studies by our team revealed the interactions between altered brain structures and elevated serum oxidative stress [24, 25]. A related important question that has yet to be clearly answered by existing research is whether peripheral inflammation affects these vulnerable areas and, if so, whether it disrupts cognition-related networks, thus causing subsequent downstream degradation and functional impairment. In any case, the identification of any highly sensitive areas might be helpful in the early detection of cognitive deficits, in treatment monitoring, and in dementia progression prevention.

In the present study, we used voxel-based morphometry (VBM) and resting state functional MRI (rs-fMRI) to evaluate the effects of systemic oxidative stress on brain morphology and functional network alterations, respectively, in PD patients with different cognitive statuses. More specifically, the study’s aims were (1) to evaluate the differences in systemic oxidative stress and GM volume changes in PD patients and healthy controls, (2) to investigate the vulnerable areas and associated functional networks in the brain that are highly sensitive to systemic oxidative parameters, and (3) to evaluate the relationships between cognitive impairment and the structural and functional integrity of the networks of the vulnerable areas.

## Methods

This prospective study enrolled 41 patients who were diagnosed with idiopathic PD from December 2010 to May 2015 according to the United Kingdom Brain Bank criteria [26] by an experienced neurologist from our hospital. For comparison, 29 sex- and age-matched healthy volunteers were recruited as a normal control (NC) group. All the participants in both groups had no other history of neurologic or psychiatric illness. All the evaluations for the PD patients, including the evaluations of clinical disease status, the MRI studies, and the neuropsychological tests, were initially assessed in the OFF-medication state achieved by the withdrawal of dopaminergic medications 12 to 18 h before testing. The Chang Gung Memorial Hospital Ethics Committee approved the study, and all of the participants provided written informed consent.

Each patient’s disease severity and cognitive functional status were evaluated using the Unified Parkinson’s Disease Rating Scale (UPDRS), the modified Hoehn and Yahr Staging Scale, the Schwab and England Activities of Daily Living Scale, and the MiniMental State Examination (MMSE). Neuropsychological evaluations of five cognitive domains (i.e., attention and working memory, executive, language, memory, and visuospatial) were conducted using subtests from the Cognitive Ability Screening Instrument [27] and the Wechsler Adult Intelligence Scale-III [28].

All the patients were classified as having PDN, PDMCI, or PDD. PDMCI was defined according to the Movement Disorder Society Task Force Guidelines [1]. Cognitive impairment was defined as a score 1.5 SD below the normative mean in each of the domains [29]. PDN was defined as less than two domains of cognitive impairment. PDMCI was defined as one score at − 1.5 SD in each of two or more domains but without dementia. PDD was defined as impairment in more than one cognitive domain with an MMSE score of less than 26 [30]. The analyses of the five cognitive function domains were used with the average Z-score of all the subtest scores in each domain.

Blood samples were drawn by venipuncture on the same day as the MRI study and neuropsychological testing were conducted. Systemic inflammation was evaluated in terms of the percentage of apoptotic peripheral leukocytes and the levels of cellular adhesion molecules. The level of leukocyte apoptosis was assessed using APO 2.7-phycoerythrin (PE). The quantities of cellular adhesion molecules were expressed as the mean fluorescence intensity of anti-LFA-1 or anti-macrophage-1 antigen (Mac-1) antibody-positive leukocytes. All the leukocytes and their subtypes were analyzed by flow cytometry. The statistical analyses were performed using the Statistical Package for Social Sciences (SPSS, version 22, SPSS Inc. Chicago, IL, USA). The group differences were compared by analysis of covariance (ANCOVA), with age and sex controlled for as potential confounding variables. Statistical significance was set at Bonferroni corrected *P* < 0.05.

For each subject, an MRI scan was performed using a 3.0 Tesla whole-body GE Signa MRI system equipped with an eight-channel head coil. A 3D high-resolution T1-weighted anatomical image was acquired using an inversion recovery fast spoiled gradient-recalled echo pulse sequence (TR/TE/inversion time = 9.5/3.9/450 ms; flip angle = 20°; FOV = 256 mm; matrix size = 512 × 512). A resting state functional image was also acquired using 300 contiguous echo planar imaging whole-brain functional scans (TR  =  2 s, TE  =  30 ms, FOV  =  240 mm, flip angle 80°, matrix size 64 × 64, thickness  =  4 mm). During the resting experiment, the scanner room was darkened and the participants were instructed to relax, with their eyes closed, without falling asleep. All patients were in the OFF-dopaminergic-medication state during the MRI scans.

T1-weighted structural MRI data were analyzed using VBM and the Statistical Parametric Mapping software program (SPM12, Wellcome Institute of Neurology, University College London, UK, http://www.fil.ion.ucl.ac.uk/spm/) with default settings. All native space T1-weighted structural MRI scans were segmented into GM, white matter (WM), and cerebrospinal fluid (CSF) components. During normalization, the GM and WM images were affine-registered to the tissue probability maps in the Montreal Neurological Institute standard space. The resulting GM tissue segment images were transformed by the DARTEL registration procedure and were smoothed with a Gaussian kernel of full width at half maximum of 8  mm. The final GM images were analyzed within the framework of a full factorial design, whereas ANCOVA was performed with age, sex, and total intracranial volume (TIV) as covariates to investigate the gray matter volume (GMV) differences between the PD and control groups. The resulting statistical inferences were considered significant if the cluster-level family-wise error corrected *P* value was < 0.05. For the correlation analyses, whole-brain voxel-based multiple regression analysis with age and gender controlled for was used to identify the GM vulnerable areas associated with significant oxidative stress markers in PD.

The rs-fMRI data were preprocessed using the SPM software program and the Data Processing Assistant for Resting-State fMRI software program. After normalization and smoothing by SPM, the waveform of each voxel was finally used for removal of the linear trends of time courses and for temporal band-pass filtering (0.01 to 0.08 Hz) [31]. For network construction of the vulnerable areas associated with oxidative stress, the GM regions previously identified as vulnerable to any significant levels of oxidative stress markers were set as seeds. The voxel-wise FC analyses of the four groups were performed by computing the temporal correlations between the mean time series of each seed and the time series of each voxel within the whole brain. The correlation coefficients of each voxel were normalized to z-scores with Fisher’s r-to-z transformation. A head movement parameter, the mean frame-wise displacement (FD), which indexes the volume-to-volume changes in head position, was also assessed for each participant. There was no significant difference between the NC and PD groups or among the PD subgroups.

To test whether the changes in FC patterns were associated with the underlying structural atrophy, the GMV values of the four groups were calculated under the intrinsic connectivity networks of the healthy controls connecting to the vulnerable areas. ANCOVAs with age, sex, and TIV (only for GMV) as the covariates were then used to compare the groups in terms of the mean GMV and functional connectivity correlation coefficient (fc-CC) values of the network area. The intergroup differences in the seed-based functional connectivity to the vulnerable areas were tested using the voxel-wise two-sample *t* test embedded in SPM. Analyses of the correlations between the fc-CC and GMV of the network and the correlations among the fc-CC, disease severity, and cognitive impairment were also performed, with the former analyses controlling for TIV and the latter analyses controlling for age and sex. The cluster size was determined over 1000 Monte Carlo simulations using the AlphaSim program distributed with the REST software tool (http://resting-fmri.sourceforge.net/). A corrected significance level of *P* = 0.01 was obtained by a combined threshold of *P* = 0.01 for each voxel and an extent threshold of 43 voxels (cluster size > 1161 mm^3^). The threshold for statistical significance was set at *P* < 0.05.

## Results

### Clinical characteristics and oxidative parameters among groups

The demographic characteristics and oxidative parameters of the 41 PD patients and 29 healthy volunteers are shown in Table 1. There were no significant differences in age, sex, and years of education between the NC group and overall PD group. However, the PDD subgroup had, on average, significantly fewer years of education, higher MMSE and UPDRS I scores, and higher medication doses. Meanwhile, the apoptosis percentage and the LFA-1 levels of monocytes and lymphocytes were significantly higher in the PDN subgroup than in the controls (Bonferroni corrected *P* < 0.05) (Table 1 and Additional file 1: Figure S1). The apoptosis percentage of monocytes and especially lymphocytes were still significantly higher in the PDN subgroup after removal of outliers (Additional file 2: Table S1).

**Table 1:** Demographic characteristics of the PD patients and normal controls

	Normal group (*n* = 29)	Patients with PD (*n* = 41)			*F*†	*P*† value
		PDN (*n* = 16)	PDMCI (*n* = 13)	PDD (*n* = 12)		
Sex (male/female)	13/16	8/8	7/6	6/6		0.954
Age	62.17 ± 4.82	61.44 ± 7.33	61.54 ± 7.52	64.50 ± 5.65	0.690	0.561
Education	9.93 ± 4.05	11.94 ± 4.17 #	9.08 ± 3.62	5.67 ± 4.01 #	5.169	*0.003†*
Disease duration		2.33 ± 1.38 #	2.44 ± 2.19	5.65 ± 5.32 #	4.340	*0.020†*
Medicine dose		261.38 ± 151.91 #	413.25 ± 283.20	512.75 ± 317.41 #	3.739	*0.034†*
MMSE		26.69 ± 3.59 #	27.31 ± 1.55 §	19.75 ± 3.60 #§	21.416	*0.000†*
UPDRS I		2.69 ± 2.24 #	3.00 ± 2.61	5.08 ± 3.34 #	3.722	*0.034†*
UPDRS II		9.00 ± 6.36	10.54 ± 9.00	13.33 ± 9.38	0.843	0.439
UPDRS III		18.06 ± 13.24	26.00 ± 17.92	31.50 ± 19.94	2.042	0.145
UPDRS total		29.75 ± 20.49	39.54 ± 28.60	49.92 ± 31.25	1.832	0.175
H&Y score		1.94 ± 1.24	2.27 ± 1.07	2.54 ± 1.01	0.710	0.499
S&E score		83.75 ± 18.93	83.08 ± 21.36	77.50 ± 18.65	0.300	0.743
Oxidation parameters						
Monocyte LFA-1	13.21 ± 4.34 ¤*	17.83 ± 5.58 ¤	17.67 ± 6.20	18.69 ± 6.02*	4.562	*0.006†*
Lymphocyte LFA-1	12.31 ± 3.10 ¤	15.37 ± 4.00 ¤	14.72 ± 1.78	14.43 ± 3.17	4.250	*0.008†*
Granulocyte LFA-1	5.42 ± 1.34	6.11 ± 1.17	6.15 ± 0.72	6.29 ± 1.69	1.841	0.149
Monocyte Mac-1	40.26 ± 30.28	49.69 ± 19.87	63.75 ± 53.22	52.90 ± 28.68	1.575	0.204
Lymphocyte Mac-1	9.91 ± 1.89	10.03 ± 2.66	10.94 ± 5.24	10.58 ± 2.37	0.477	0.699
Granulocyte Mac-1	41.42 ± 15.35	49.66 ± 20.81	56.41 ± 25.01	50.71 ± 27.27	1.921	0.135
Monocyte APO2.7 (%)	2.28 ± 1.30 ¤	8.00 ± 9.82 ¤	4.13 ± 3.44	4.83 ± 4.52	4.361	*0.007†*
Lymphocyte APO2.7 (%)	0.48 ± 0.38 ¤	1.04 ± 0.71 ¤	0.56 ± 0.38	0.62 ± 0.41	5.305	*0.002†*
Granulocyte APO2.7 (%)	0.82 ± 0.58	1.03 ± 0.57	1.17 ± 1.19	1.11 ± 0.79	0.799	0.499

Data are presented as mean ± standard deviation. Sex data were compared by Pearson chi-square test. Age data were compared by independent *t* test. Education year, oxidation parameters, clinical severity score, medication, and cognitive function data were compared by analysis of covariance (ANCOVA) after controlling for age and sex. *F*† and *P*† represent the comparison amounts of the PDN, PDMCI, and PDD patients and the normal control group, controlling for age and sex, with Bonferroni correction

¤ Significant differences between NC and PDN; * Significant differences between NC and PDD; # Significant differences between PDN and PDD; § Significant differences between PDMCI and PDD; †Significant differences among groups

### Changes of gray matter volume in PD subgroups

Compared with the NC group, the PDD subgroup showed significantly diffuse GMV loss throughout the whole brain; the PDMCI and PDN subgroups showed significantly smaller GMV values (Additional file 3: Figure S2). There was no brain region in which the PD subgroups had significantly greater GMV than the NC group.

### Vulnerable areas associated with oxidative parameters in PD group

**Fig. 1:**
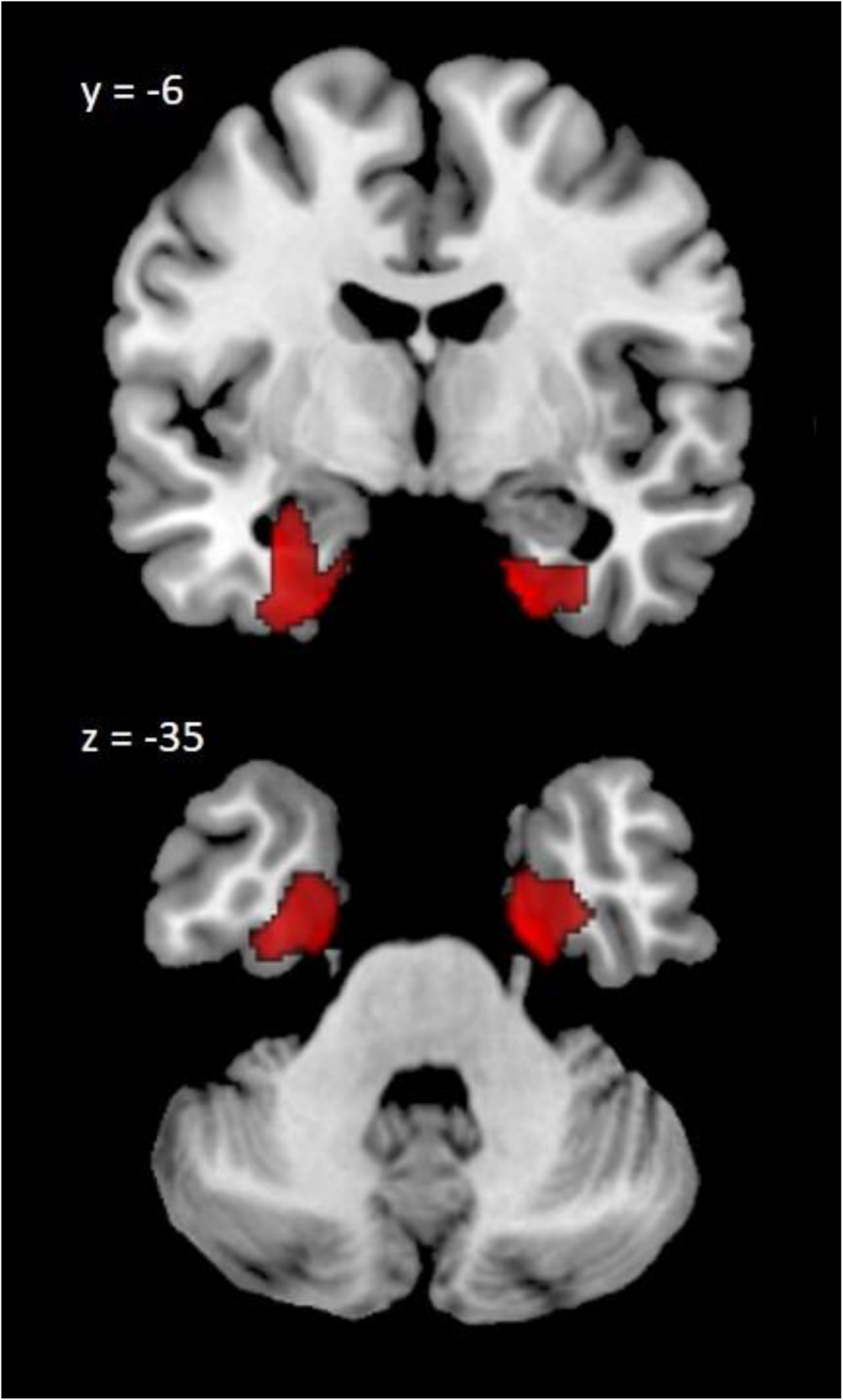
Neuronal vulnerable areas associated with lymphocyte. Bilateral mesial temporal lobes (MTLs, MNI space[*x*,*y*,*z*]: [− 32,− 6,− 29] and [18,− 2,− 35]) were identified as the vulnerable areas associated with lymphocyte apoptosis in PD patients (corrected *P* < 0.005, cluster > 288) (controlling for age, sex, and TIV)

Using whole-brain voxel-based correlation analysis for the PD group, the bilateral MTLs (MNI space[*x*,*y*,*z*]: [− 32,− 6,− 29] and [18,− 2,− 35]) were identified as the vulnerable areas associated with lymphocyte apoptosis (*P* < 0.005, cluster > 288) (Fig. 1). This meant that the GMV of the MTL vulnerable areas was correlated with the lymphocyte apoptosis marker in the PD group. No vulnerable areas associated with other oxidative parameters in the PD brains were identified.

### Analysis of intrinsic connectivity networks connected to the vulnerable areas

**Fig. 2:**
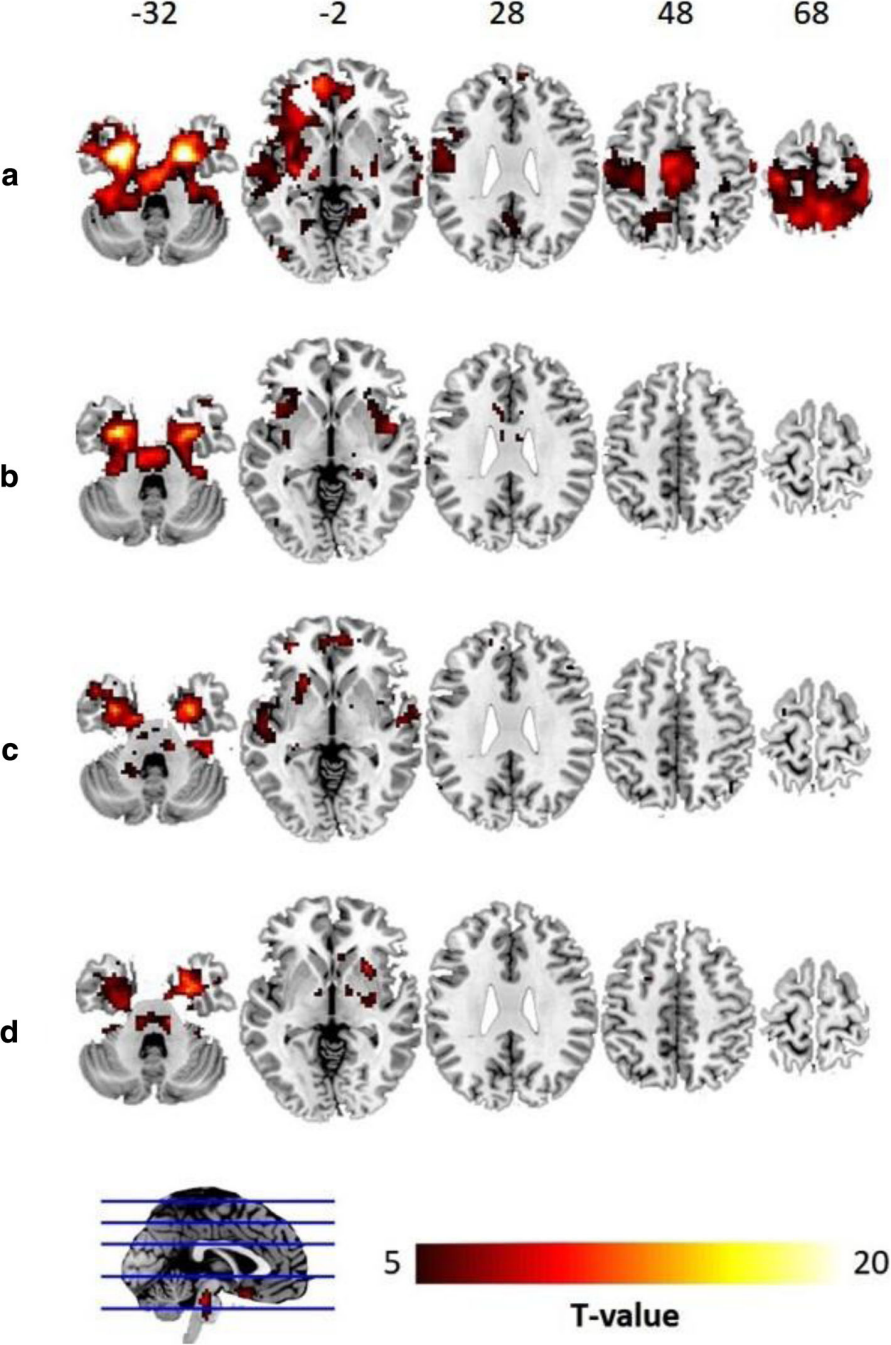
The MTL functional network. The MTL functional network seeded by the epicenters vulnerable to lymphocyte apoptosis in the NC (**a**), PDN (**b**), PDMCI (**c**), and PDD (**d**) groups. The intrinsic MTL functional network of healthy subjects (FEW corrected *P* < 0.01) is shown in **a** and reveals connectivity between the temporal and frontal lobes and other regions of the neocortex. Disrupted MTL functional networks, however, are shown for the PD patients, especially for the PDD subgroup

The intrinsic connectivity network of the vulnerable area was constructed by using the bilateral MTLs as seed regions. We found that the functional connectivity with the bilateral MTL vulnerable areas became limited and disrupted in the PD patients (Fig. 2). The MTL functional network profile derived from the healthy subjects resembled the PD-associated GM atrophy pattern (Fig. 2 and Additional file 3: Figure S2). However, there were no significant fc-CC value differences of the bilateral MTL functional network among the PD subgroups.

**Fig. 3:**
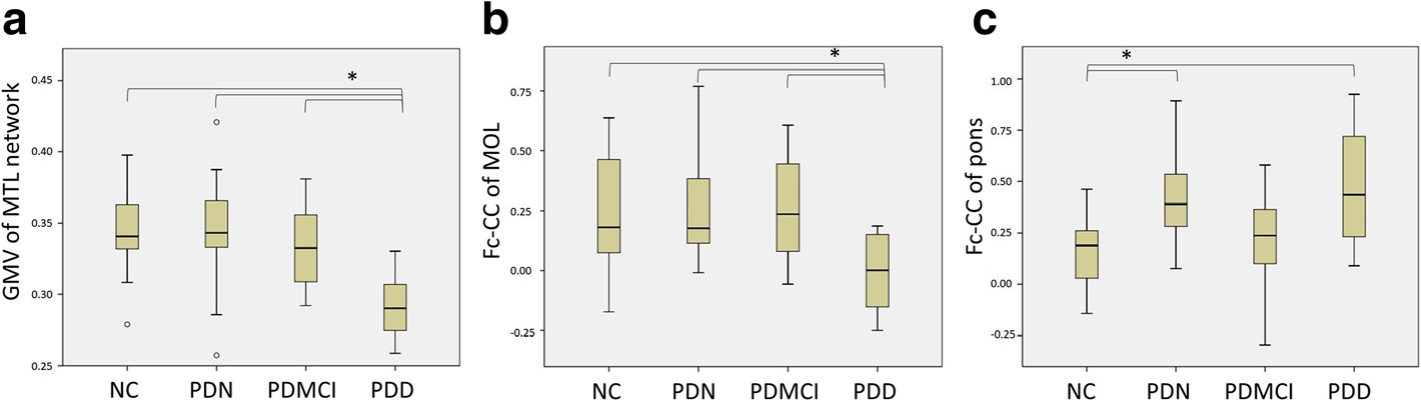
The structural and functional alteration in the MTL network. (**a)** Comparison of GMVs under the intrinsic MTL functional network among the NC group and PD subgroups revealed significant atrophy in the PDD subgroup. (**b**) The functional connectivity between the MTL and middle occipital lobe (MOL) within the MTL functional network was significantly decreased in the PDD subgroup, (**c**) but the functional connectivity between the MTL and pons was significantly increased in the PDN and PDD subgroups

By using the MTL functional network of the healthy controls as a mask, we found that the GMVs underlying the MTL functional network were significantly smaller in the PDD subgroup than in the NC group and other PD subgroups (*P* < 0.001) (Fig. 3a).

Finally, the two-sample *t* test conducted to compare all the PD patients and the NC group showed significant changes of the fc-CC values in the middle occipital lobe (MOL) and pons within the MTL functional network (Additional file 4: Figure S3). The MOL fc-CC value was significantly decreased in the PDD subgroup compared with the NC group and other PD subgroups. Otherwise, the fc-CC value of the pons was significantly increased in the PDN and PDD subgroups compared with the NC group (Fig. 3b, c).

### Relationship between functional connectivity and gray matter volume of MTL functional network

Within the MTL functional network, we found that the reduced GMV was associated with decreased fc-CC (*r* = − 0.332, *P* = 0.036) values in the PD patients. Disruptions to the MTL network were seen in both its architecture and functional connectivity, and these architectural and functional disruptions were associated with each other.

### Relationships among disease severity, cognitive function, and the MTL functional network

Correlation analyses were conducted to evaluate the relationships among the disease severity, cognitive function, and fc-CC values. In general, the aggregated disease severity and cognitive impairment were associated with lower functional connectivity in the PD patients. Due to the exploratory design of the study, we performed the correlation analyses with and without controlling clinical parameters, such as education year, disease duration, and medication.

**Fig. 4:**
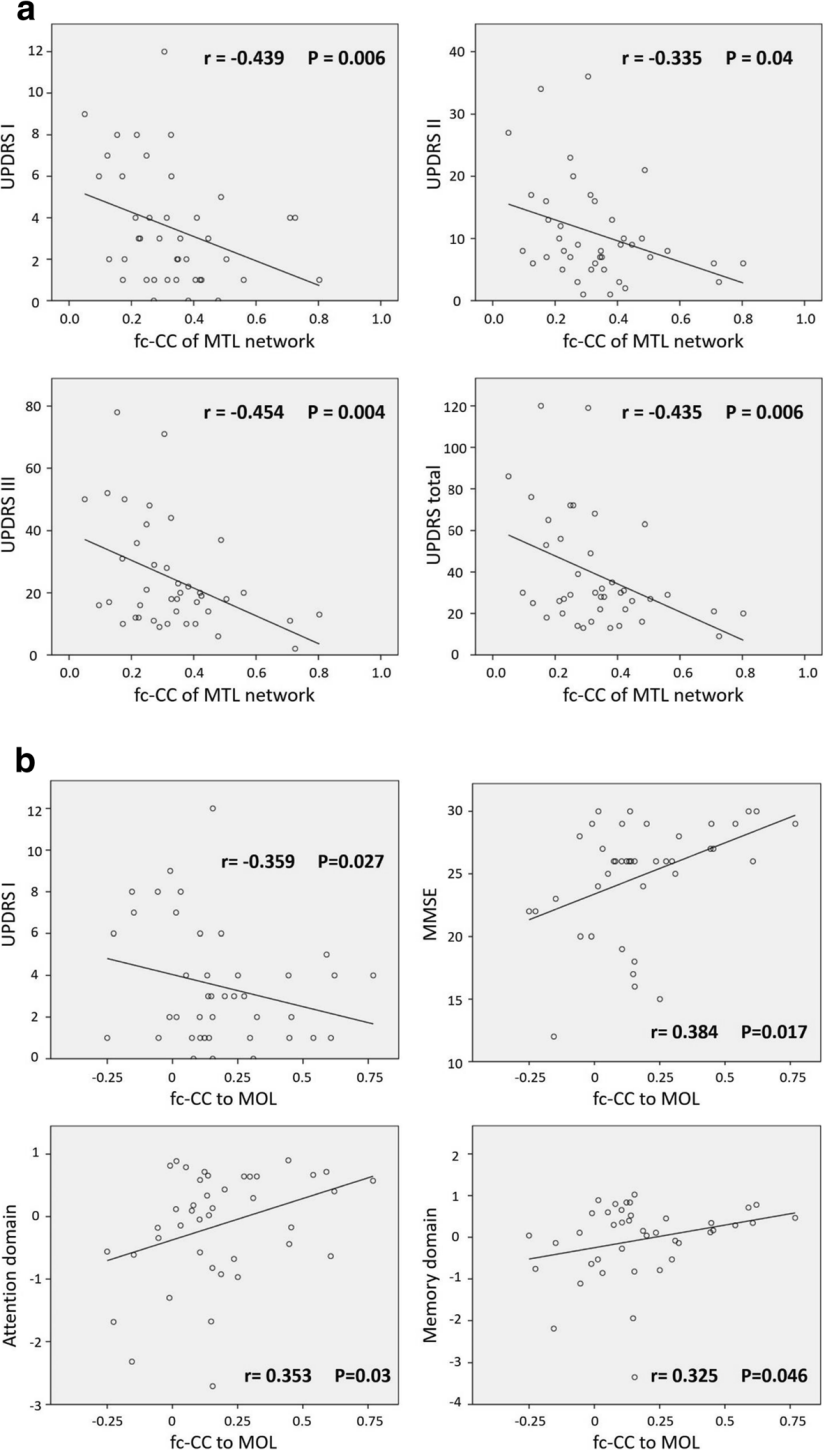
**a** The correlation between disease severity and functional connectivity within the MTL functional network. The reduced fc-CC values of the MTL functional network were associated with worsening clinical symptoms, including UPDRS part I (mentation and mood) and part III (motor) and total scores (controlling for age and sex, Bonferroni corrected *P* < 0.05). **b** The correlation between cognition and functional connectivity within the MTL functional network. The reduced fc-CC values of the middle occipital lobe were associated with worsening cognitive impairment, including UPDRS part I (mentation and mood), MMSE, attention, and memory function scores (controlling for age and sex, Bonferroni corrected *P* < 0.05)

With controlling age and gender only, the reduced fc-CC values of the MTL functional network were associated with greater disease severity, including in terms of the UPDRS part I (*r* = − 0.439, *P* = 0.006) and III (*r* = − 0.454, *P* = 0.004) and total scores (*r* = − 0.435, *P* = 0.006) (Fig. 4a). In addition, the reduced fc-CC values between the MOL and MTL of the PD patients were also associated with greater disease severity as indicated by the UPDRS I (*r* = − 0.359, *P* = 0.027) and MMSE scores (*r* = 0.384, *P* = 0.017), as well as with greater cognitive impairment in the attention (*r* = 0.353, *P* = 0.03) and memory (*r* = 0.325, *P* = 0.046) domains (Fig. 4b).

In further analyses with education year, disease duration and medication as covariates, the reduced fc-CC values of the MTL functional network were less significantly associated with greater disease severity, including in terms of the UPDRS part I (*r* = − 0. 381, *P* = 0.024) and III (*r* = − 0.406, *P* = 0.016) and total scores (*r* = − 0.385, *P* = 0.022). The associations of the increased pons fc-CC values with greater disease severity as indicated by the UPDRS I (*r* = 0.404, *P* = 0.016) and MMSE scores (*r* = − 0.388, *P* = 0.021), as well as with greater cognitive impairment in the attention (*r* = − 0.466, *P* = 0.005) and memory (*r* = − 0.471, *P* = 0.004) domains were emphasized. But the associations between the fc-CC values of the MOL and disease severity as indicated by the UPDRS I (*r* = − 0.320, *P* = 0.061) and MMSE scores (*r* = 0.411, *P* = 0.014), as well as greater cognitive impairment in the attention (*r* = 0.330, *P* = 0.053) and memory (*r* = 0.327, *P* = 0.055) domains were weakened.

## Discussion

In the comparison between the PD patients and healthy controls, the PD patients presented with higher levels of systemic oxidative stress, including increased apoptosis percentages and increased LFA-1 values. Compared with the NC group, the PD patients had significantly lower GMVs with diffuse atrophy patterns, especially the PDD patients. The MTL atrophy was used to identify the MTLs as the vulnerable areas associated with lymphocyte apoptosis, indicating their relationship with systemic oxidative stress. In addition, we also found deterioration of the structural and functional network integrity of the MTL network, a finding which further associated the network with the progression of disease severity and cognitive deficits. These associations may reflect the possible key role of lymphocyte apoptosis in the GMV atrophy and functional connectivity alterations seen in the development of PD.

Increased apoptosis of lymphocytes and monocytes and increased expression of LFA-1 might support the view that there is only one primary pathophysiology of PD: abnormal systemic oxidative stress and the acceleration of peripheral immune cell infiltration into the brain. Neurodegeneration in PD associated with systemic inflammation via various pathways has been thoroughly discussed [12, 13, 16–18, 24, 25]. Several previous studies showed that higher percentages of peripheral apoptotic lymphocytes [32] and the CNS infiltration of lymphocytes and monocytes contribute to neurodegeneration [22, 33, 34]. The activation of LFA-1 has been shown to mediate the recruitment of peripheral lymphocytes and monocytes into the PD brain [22]. Although researchers have faced some difficulty in verifying the direct interactions between systemic oxidative stress and neuroinflammation in PD subjects, the infiltration of peripheral immune cells into the SN of animal PD models [13] and the association between leukocyte apoptosis and striatal dopamine neuron loss [16] constitute indirect forms of evidence provided by previous studies. Current study may further provide an indirect evidence of peripheral inflammation communicating with the brain with immune cells in PD.

In terms of comparisons among the PD subgroups, our data revealed that the PDN group had a higher level oxidative stress than the other PD subgroups. At the same time, the trajectory of oxidative stress presented double peaks in the PDMCI and PDD subgroups (Table 1 and Additional file 1: Figure S1). To the best of our knowledge, there have been no previous studies discussing the changes in oxidative stress in PD in terms of different cognitive functions. The trajectory of oxidative stress seen in the present study, however, was similar to that of the hypothetical model of a dual peak of microglial activation in Alzheimer’s disease proposed by Fan et al. [35], wherein ramified microglia transform into anti-inflammatory and pro-inflammatory phenotypes. Dynamic changes to the peripheral immune system in mild (early) and severe (late) cognitive status might also reflect the progression of neurodegeneration in a non-cell-autonomous fashion with respect to neurons [35]. Although we could not verify the phenotypes of leukocytes in the present study, the higher immune response seen in PD without dementia might suggest a stage with higher levels of CNS pro-inflammatory phenotypes, such as increased apoptosis percentage and LFA-1 values.

Although GMV was diffusely and significantly reduced in all the PD patients relative to the normal controls in the present study, only the GMV reductions in the bilateral MTLs were significantly associated with peripheral lymphocyte apoptosis accompanied with cognitive impairment. The high susceptibility of the MTL suggests its role as an important area in maintaining cognitive ability in PD. The cell-autonomous factors of neuron govern both for and against the influence of peripheral immune cells [36], might determine the different sensitivity over different brain regions. The MTL is known as a very sensitive area for chronic stress and hypoxia conditions [37–39] and is among the brain areas most easily affected in Alzheimer’s disease [40]. The neurons in the MTL have large cell surfaces and high-energy requirements, which makes these neurons more prone to receiving multiple forms of oxidative stress [40]. Our whole-brain VBM-based correlation analysis with lower presumptions further highlights the reliability of the observed findings.

We found that the topographic distribution of the MTL functional network derived from the healthy subjects was partially overlaid with the key components of the cholinergic pathways from the BNM network to the frontal and temporal cortices [3]. The BNM network is responsible for maintaining fronto-executive and memory functions [41]. Relatedly, decreased cholinergic neurons in the frontal and temporal lobes are believed to be associated with the cognitive impairments seen in PDMCI and PDD [3]. Alterations of MTL intra-network functional connectivity between the MTL and MOL were also found. Meanwhile, attention and memory impairments were observed to be associated with decreased functional connectivity between the MTL and MOL. Interestingly, the MTL-pons connectivity in the PDMCI subgroup was lower than that for the other PD subgroups. The cause of this presentation is still uncertain and requires further investigation and research.

We also found progressive underlying structural deficits of the MTL network in PDN, PDMCI, and PDD. Those temporo-spatial degradation results reflect the functional and anatomical proximity of the MTL network to presumed disease vulnerable areas, and highlight the value of systemic lymphocyte levels in the evaluation of the network degenerative mechanisms in PD. According to the network degeneration hypothesis [11], the different pathological molecules, such as mis-folded proteins, target different specific brain vulnerable areas and their networks. These selective forms of neuronal vulnerability may be related to the weakening of synaptic convergence zones, the loss of retrograde growth factor supplement, the transsynaptic spread of mis-folded protein, and other factors related to network destabilization [11]. The concordance between disease-related atrophy and healthy intrinsic functional connectivity reflects the network-driven neuronal vulnerability. However, the accumulation of mis-folded alpha-synuclein in Lewy bodies and Lewy neurites, a major hallmark of PD [42], was not evaluated in the present study. Its effects and interactions with systemic inflammation in the MTL were thus not determined, and a further validation study should therefore be conducted.

This study has some limitations. First, it was a small-sized and cross-sectional study, which limits any interpretations that can be made from it regarding the trajectory of oxidative stress markers, the structural or functional alterations as the disease progresses, and any causal relationships among the above parameters. Second, multiple comparison correction was not applied for the correlational analyses due to the small sample sizes after subgrouping, the exploratory nature of the study, and the possibility of overcorrection owing to the high collinearity of the studied clinical and cognitive parameters. As such, the correlational analyses might be underpowered, and the significance of the associated results should be interpreted cautiously. Third, we did not verify the phenotypes of peripheral immune cells. As such, only successful therapeutic trials targeting those oxidative stress makers, their upstream causes, or their downstream effects will confirm that PD are indeed caused by processes related to immune cell infiltration. Manipulations of the systemic immune system and mis-folded proteins and evaluations of their effects in PD animal models might help to verify the role of the MTL in cognition in the future.

## Conclusion

In terms of cognitive impairment, this study demonstrates that the neuronal vulnerable areas associated with lymphocyte infiltration are located in the bilateral MTLs, with such infiltration resulting in the disruption of both architectural and functional connectivity. Lymphocyte-associated MTL atrophy may thus represent the possible etiology of PD.

## Additional files

Additional file 1: **Figure S1.** Systemic oxidative stress in PD. Systemic oxidative stress was significantly increased in the PD patients. Compared with the NC group, the LFA-1 levels of monocytes were significantly higher in the PDD subgroup. Furthermore, the apoptosis percentage and the LFA-1 levels of monocytes and lymphocytes were significantly higher in the PDN subgroup than in the controls. (MFI: mean fluorescence intensity) (*Bonferroni corrected *P* < 0.05). (DOCX 376 kb)

Additional file 2: **Table S1.** Oxidation parameters of the PD patients and normal controls after removal of outliers. (DOCX 18 kb)

Additional file 3: **Figure S2.** The alternation of gray matter volume in PD. Gray matter atrophy patterns in the PD subgroups compared with the NC group and with each other. The PDD subgroup showed significantly diffuse GMV loss compared with the PDMCI and PDN subgroups, as well as the NC group. The PDN and PDMCI subgroups showed only small areas of GMV loss compared with the NC group, and there was no significant difference between them. (Corrected *P* < 0.005, cluster> 350). (DOCX 210 kb)

Additional file 4: **Figure S3.** The alternation of functional connectivity within the MTL functional network in PD. The two-sample *t* test between all the PD patients and the NC group showed significant changes of the fc-CC values in the middle occipital lobe (MOL) and pons within the MTL functional network. (Corrected *P* < 0.001, cluster = 19). (DOCX 74 kb)

## Abbreviations

ANCOVA: Analysis of covariance
BBB: Blood-brain barrier
BNM: Basal nucleus of Meynert
CNS: Central nervous system
CSF: Cerebrospinal fluid
fc-CC: Functional connectivity correlation coefficient
GM: Gray matter
GMV: Gray matter volume
LFA-1: Lymphocyte function-associated antigen 1
Mac-1: Macrophage-1 antigen
MMSE: MiniMental State Examination
MOL: Middle occipital lobe
MTLs: Mesial temporal lobes
NC: Normal control
PD: Parkinson’s disease
PDD: PD with dementia
PDMCI: PD with mild cognitive impairment
PDN: PD with normal cognition
PE: Phycoerythrin
rs-fMRI: Resting state functional MRI
SN: Substantia nigra
SPM: Statistical Parametric Mapping software program
SPSS: Statistical Package for Social Sciences
TIV: Total intracranial volume
UPDRS: Unified Parkinson’s Disease Rating Scale
VBM: Voxel-based morphometry
WM: White matter
