# Supplementary figures and images for: Interaction of systemic oxidative stress and mesial temporal network degeneration in Parkinson’s disease with and without cognitive impairment
Source: J Neuroinflammation. 2018 Sep 26;15:281. doi: 10.1186/s12974-018-1317-z (PMC6158841; doi:10.1186/s12974-018-1317-z)

**Figure S1**


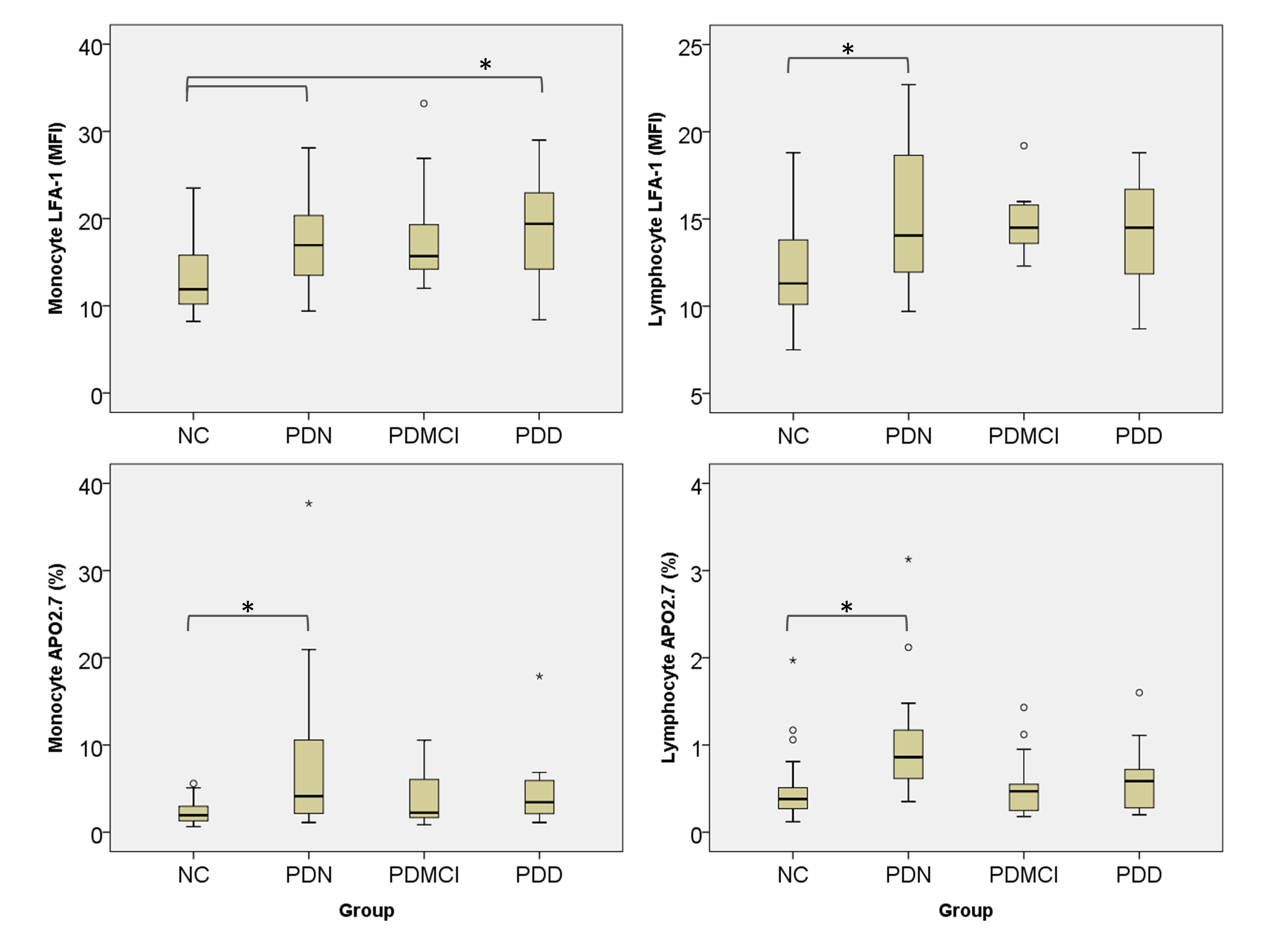

Supplement: Supplementary file 1 — Figure S1. Systemic oxidative stress in PD. Systemic oxidative stress was significantly increased in the PD patients. Compared with the NC group, the LFA-1 levels of monocytes were significantly higher in the PDD subgroup. Furthermore, the apoptosis percentage and the LFA-1 levels of monocytes and lymphocytes were significantly higher in the PDN subgroup than in the controls. (MFI: mean fluorescence intensity) (*Bonferroni corrected P < 0.05). (DOCX 376 kb) [file 12974_2018_1317_MOESM1_ESM.docx]

**Figure S2**


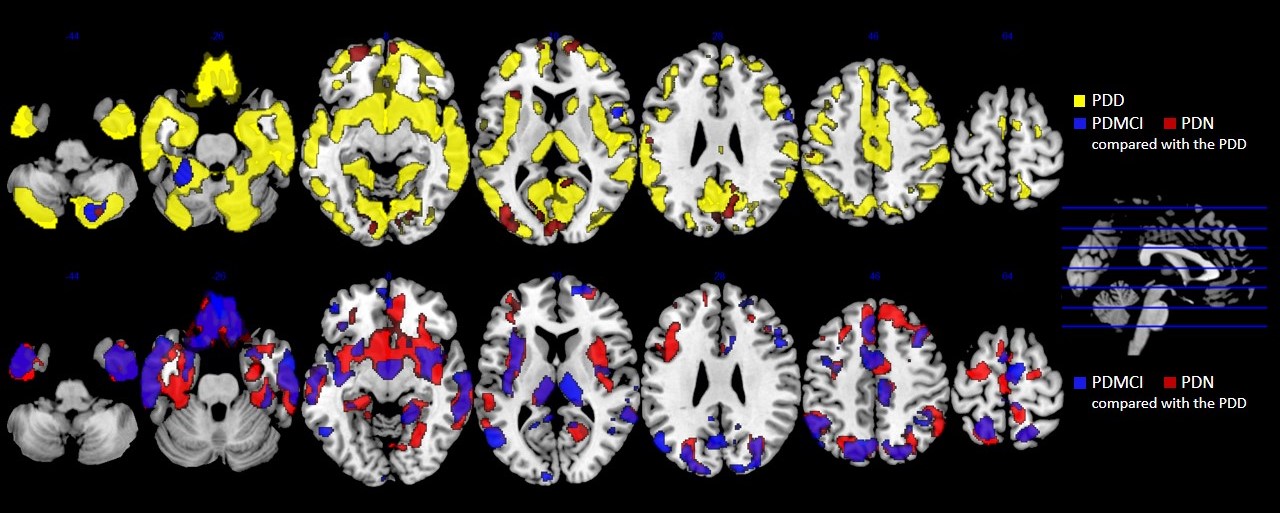

Supplement: Supplementary file 3 — Figure S2. The alternation of gray matter volume in PD. Gray matter atrophy patterns in the PD subgroups compared with the NC group and with each other. The PDD subgroup showed significantly diffuse GMV loss compared with the PDMCI and PDN subgroups, as well as the NC group. The PDN and PDMCI subgroups showed only small areas of GMV loss compared with the NC group, and there was no significant difference between them. (Corrected P < 0.005, cluster> 350). (DOCX 210 kb) [file 12974_2018_1317_MOESM3_ESM.docx]

**Figure S3:**


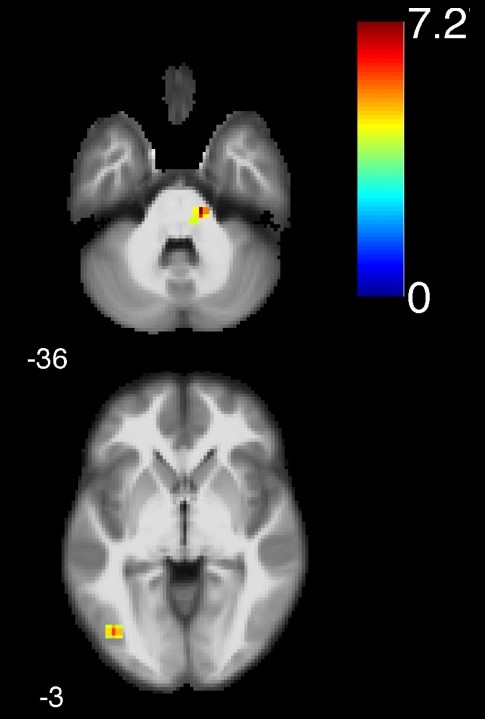

Supplement: Supplementary file 4 — Figure S3. The alternation of functional connectivity within the MTL functional network in PD. The two-sample t test between all the PD patients and the NC group showed significant changes of the fc-CC values in the middle occipital lobe (MOL) and pons within the MTL functional network. (Corrected P < 0.001, cluster = 19). (DOCX 74 kb) [file 12974_2018_1317_MOESM4_ESM.docx]
