# Supplementary material for: Interaction of systemic oxidative stress and mesial temporal network degeneration in Parkinson’s disease with and without cognitive impairment
Source: J Neuroinflammation. 2018 Sep 26;15:281. doi: 10.1186/s12974-018-1317-z (PMC6158841; doi:10.1186/s12974-018-1317-z)
Supplement: Supplementary file 2 — Table S1. Oxidation parameters of the PD patients and normal controls after removal of outliers. (DOCX 18 kb) [file 12974_2018_1317_MOESM2_ESM.docx]

**Table S1:** Oxidation parameters of the PD patients and normal controls after removal of outliers.

|  | Normal group  (n=26) | Patients with PD (n=33) | | | F† | P† value |
| --- | --- | --- | --- | --- | --- | --- |
|  |  | PDN (n=13) | PDMCI (n=10) | PDD (n=10) |  |  |
| Oxidation parameters | | | | | | |
| Monocyte LFA-1 | 13.42 ± 4.53 * | 17.62 ± 5.20 | 16.37 ± 6.36 | 19.09 ± 6.35 * | 3.864 | ***0.014*** |
| Lymphocyte LFA-1 | 12.54 ± 3.17 | 14.95 ± 4.16 | 14.40 ± 1.05 | 14.02 ± 3.34 | 2.431 | ***0.075*** |
| Monocyte APO2.7 (%) | 2.02 ± 1.03 ¤ | 4.11 ± 3.41 ¤ | 2.91 ± 2.42 | 3.40 ± 1.91 | 3.014 | ***0.038*** |
| Lymphocyte APO2.7 (%) | 0.38 ± 0.46 ¤ | 0.79 ± 0.66 ¤§# | 0.42 ± 0.74§ | 0.48 ± 0.75# | 9.049 | ***< 0.001*** |

Data are presented as mean ± standard deviation. Oxidation parameters were compared by analysis of covariance (ANCOVA) after controlling for age and sex. F† and P† represent the comparison amounts of the PDN, PDMCI, and PDD patients and the normal control group, controlling for age and sex, with Bonferroni correction.

¤ Significant differences between NC and PDN; * Significant differences between NC and PDD; § Significant differences between PDN and PDMCI; # Significant differences between PDN and PDD.
